# High mobility group box 1 promotes sorafenib resistance in HepG2 cells and in vivo

**DOI:** 10.1186/s12885-017-3868-2

**Published:** 2017-12-15

**Authors:** Yinzong Xiao, Lunquan Sun, Yongming Fu, Yan Huang, Rongrong Zhou, Xingwang Hu, Pengcheng Zhou, Jun Quan, Ning Li, Xue-Gong Fan

**Affiliations:** 1 Hunan Key Laboratory of Viral Hepatitis, Department of Infectious Diseases, Xiangya Hospital, Central South University, Changsha, 410008 China; 2 Center for Molecular Medicine, Xiangya Hospital, Key Laboratory of Molecular Radiation Oncology of Hunan Province, Central South University, Changsha, 410008 China; 3 Department of Blood Transfusion, Xiangya Hospital, Central South University, Changsha, 410008 China

**Keywords:** HCC, HMGB1, Sorafenib resistance, Mitochondria

## Abstract

**Background:**

Primary liver cancer is a lethal malignancy with a high mortality worldwide. Currently, sorafenib is the most effective molecular-targeted drug against hepatocellular carcinoma (HCC). However, the sorafenib resistance rate is high. The molecular mechanism of this resistance has not been fully elucidated. High mobility group box 1 (HMGB1) is a multifaceted protein that plays a key role in the proliferation, apoptosis, metastasis and angiogenesis of HCC cells. In addition, HMGB1 has been suggested to contribute to chemotherapy resistance in tumours, including lung cancer, osteosarcoma, neuroblastoma, leukaemia, and colorectal cancer. This study investigated the association between HMGB1 and sorafenib resistance in HCC.

**Methods:**

HepG2 cells with HMGB1 knockdown or overexpression were generated. The efficacy of sorafenib in these cells was tested using flow cytometry and a cell counting assay. The subcellular localization of HMGB1 in HepG2 cells following sorafenib treatment was measured by western blotting and confocal microscopy. A murine subcutaneous HCC model was generated to examine the association between HMGB1 and the sensitivity of sorafenib treatment.

**Results:**

The HMGB1 knockdown cells exhibited a significantly higher apoptotic level and lower cell viability than the normal HMGB1 expressing cells following the sorafenib treatment. In addition, the cell viability observed in the HMGB1 overexpressing cells was higher than that observed in the control cells following the sorafenib intervention. Sorafenib had a better tumour inhibition effect in the HMGB1 knockdown group in vivo. The amount of mitochondrial HMGB1 decreased, while the amount of cytosolic HMGB1 increased following the exposure to sorafenib. Altogether, HMGB1 translocated from the mitochondria to the cytoplasm outside the mitochondria following the exposure of HepG2 cells to sorafenib.

**Conclusions:**

A novel potential role of HMGB1 in the regulation of sorafenib therapy resistance in HCC was observed. The knockdown of HMGB1 restores sensitivity to sorafenib and enhances HepG2 cell death, while HMGB1 overexpression blunts these effects. The translocation of HMGB1 from the mitochondria to the cytosol following sorafenib treatment provides new insight into sorafenib resistance in HCC.

**Electronic supplementary material:**

The online version of this article (10.1186/s12885-017-3868-2) contains supplementary material, which is available to authorized users.

## Background

Primary carcinoma of the liver is the second most common cause of death from cancer worldwide. Hepatocellular carcinoma (HCC) accounts for 90% of all liver cancers. HCC has a very poor prognosis, and the overall ratio of mortality to incidence is 0.95 globally [1]. Less than 30% of newly diagnosed HCC patients without surveillance are eligible for curative treatments, such as resection, transplantation, or ablation [2]. In patients with advanced HCC, sorafenib (Nexavar), which is a molecular-targeted therapy, significantly helps prolong the median survival time by approximately 3 months. Sorafenib inhibits B-RAF, vascular endothelial growth factor receptor (VEGFR), and platelet-derived growth factor receptor (PDGFR) [3]. However, only approximately 30% of advanced HCC patients benefit from sorafenib, and acquired resistance often develops within 6 months [4]. The extremely high sorafenib resistance rate has raised great concern worldwide, and the epithelial-mesenchymal transition (EMT), cancer stem cells, and tumour microenvironment may be involved [5]. However, the mechanisms underlying primary and acquired sorafenib resistance in HCC remain unclear. Currently, no other chemotherapeutic agent yields the results obtained with sorafenib; thus, understanding and overcoming sorafenib chemoresistance is critical for improving survival in advanced HCC populations [6].

The high mobility group box 1 (HMGB1) protein has been shown to play pivotal roles in HCC, including tumourigenesis, progression, invasion, metastasis, and prognosis. The mechanisms involved in the context-dependent role of HMGB1 include the regulation of cell proliferation, differentiation, cell death, inflammation and immune function in HCC [7–10].

HMGB1 is a nuclear protein that plays a role in various biological events in the nucleus, including DNA replication, repair, recombination, transcription, and genomic stability [11]. In addition to its significant nuclear role, extracellular HMGB1 is one of the most common damage-associated molecular patterns (DAMPs) with well-defined interactions with the receptor for advanced glycation end products (RAGEs) and Toll-like receptors (TLRs). The binding of HMGB1 to RAGE and TLRs affects HCC invasion, metastasis, and treatment [12, 13]. HMGB1 has also been observed in the cytosol, including the mitochondria [14], but its function in the cytoplasm remains poorly understood. Cytosolic HMGB1 may be involved in different types of cell death, and has been found to be a positive regulator of autophagy through its binding to Beclin-1 [15].

HMGB1 affects tumour growth, metastasis, and prognosis through multiple signalling pathways in cancers, including lung cancer, osteosarcoma, gastric cancer, cervical cancer, and HCC [16–19]. The mechanisms by which HMGB1 affects these biological processes vary in different malignancies. In addition, the role of HMGB1 in drug resistance in tumours, including lung cancer, osteosarcoma, neuroblastoma, leukaemia, and colorectal cancer, has attracted broad attention [20–23]. Due to its versatile role in cancer, HMGB1 has been proposed as a potential biomarker of survival and a target for cancer therapy [18, 24].

Although the role of HMGB1 in drug resistance in different malignancies has been frequently discussed, its function in anticancer therapy in HCC is not fully understood. Because HMGB1 affects multiple biological events in HCC and contributes to drug resistance in certain cancers [25], this study aims to explore the role of HMGB1 in sorafenib resistance in HCC using the HepG2 cell line and murine models.

## Methods

### Cell lines

The human hepatocarcinoma cell line HepG2 was purchased from the Cell Bank of Shanghai Academy of Science (Shanghai, China, No. TCHu 72). The cells were cultured in Dulbecco’s modified eagle medium (DMEM, Thermo Fisher Scientific Inc., USA) supplemented with 10% foetal bovine serum (FBS, Thermo Fisher Scientific Inc., USA), 100 U/mL penicillin, and 100 mg/mL streptomycin (Sangon, Shanghai, China). All cells were cultured at 37 °C in a humidified incubator with 5% CO_2_ concentration.

### Establishment of stable HMGB1-overexpressing cells and HMGB1 knockdown cells

shRNAs based on the HMGB1 sequence (NM_001313893.1) were designed as follows: shhmgb1, 5′-GCCCGTTATGAAAGAGAAATTTTTCAAGAGAAAATTTCTCTTTCATAACGGGTTTTTT-3′. The shHMGB1 shRNA sequences were cloned into PLVX-sh1 vectors (Clontech, Mountain View, CA, USA). Lentiviral vectors encoding the human HMGB1 gene were constructed in the overexpression vector (PLVX-ires-puro) (Clontech) and designated LV-HMGB1. The lentiviral vectors were transfected into HCC cells at a multiplicity of infection (MOI) of 30 to 50 in the presence of polybrene (8 μg/mL). At 72 h after the infection, the infected cells were treated with 1 μg/mL puromycin (Origene) for 2 weeks; then, the HMGB1 expression was detected using western blotting. Pooled populations of knockdown and overexpression cells were obtained 2 weeks after the drug selection without sub-cloning and used for the in vivo experiments.

### Reagents

Sorafenib was purchased from Santa Cruz Biotechnology, Inc. (Texas, US No. Sc-220,125). Sorafenib was dissolved in DMSO (Sigma-Aldrich) and diluted with DMEM or normal saline (NS) to the desired concentration for the in vitro and in vivo studies, respectively. The concentration of the mother liquid was 10 mM/L according to the manufacturer’s instructions. The doses chosen for the in vitro (40 mg/kg/d) and in vivo studies were based on those used in other published studies [26–29].

### Real-time polymerase chain reaction (RT-PCR)

The total mRNA expression was quantitatively analysed using the SYBR Green fluorescent-based assay (TaKaRa Bio, Otsu, Japan). The RT-PCR assays were performed using glyceraldehyde 3-phosphate dehydrogenate (GAPDH) as an internal control. Independent experiments were repeated three times for each sample, and the relative gene expression levels were analysed using the 2^-ΔΔCT^ method. The primers, which were designed by Sangon Biotech (Shanghai), were as follows: For HMGB1: forward 5′-TATGGCAAAAGCGGACAAGG-3′ and reverse 5′-CTTCGCAACATCACCAATGGA-3′ For GAPDH: forward 5′-GAGAGGGAAATCGTGCGTGAC-3′ and reverse 5′-CATCTGCTGGAAGGTGGACA-3′.

### Cell viability assay

The cell survival rates and dose-dependent curves of sorafenib were evaluated using the Cell Counting Kit-8 (CCK-8 Kit, Beyotime Biotechnology, China, No. C0038). The cells were plated at a density of 5000 cells per well in 96-well flat-bottomed plates and incubated at 37 °C in a humidified atmosphere of 5% CO_2_ for 24 h. The medium was replaced with various concentrations of sorafenib in DMEM and 10% FBS, and the cells were incubated for an additional 24–48 h. The supernatants were removed from the wells, and 10 μL/well of CCK-8 dye were added along with 200 μL/well of fresh DMEM. Absorbance was determined at 450 nm using a plate reader.

### Western blotting

The total protein was harvested and mitochondrial proteins were separated using the Mitochondria Extraction Kit (BioVision, NO. K256–25, USA). Nuclear protein was extracted using the Nucleoprotein Extraction Kit (Sangon, NO. C500009, China). For the HepG2 cells, 10 μg protein were loaded to test the whole lysate, while 30 μg protein were loaded to test the cell fractions. For the transfection of the HepG2 cells, 30 μg protein were loaded to examine the level of c-PARP, and 20 μg protein were loaded to examine the level of HMGB1. Following the SDS-PAGE, the proteins were transferred onto PVDF membranes (Sigma-Aldrich), hybridized with specific primary antibodies, and incubated with HRP-conjugated sheep anti-mouse/rabbit IgG (Cell Signaling, USA, 1:2000). The bands were visualized using an ECL Kit (Merck Millipore, Darmstadt, Germany) per the manufacturer’s instruction. HMGB1 (Abcam, USA, 1:1000), c-PARP (Cell Signaling, USA, 1:1000), GAPDH (Cell Signaling, USA, 1:1000), and Actin (Cell Signaling, USA, 1:1000) antibodies were used.

### Fluorescence-activated cell sorting (FACS) analysis

Annexin V-PI staining (KeyGen Biotech, China) was performed to measure the phosphatidylserine externalization in HepG2 cells transfected with different siRNAs or plvx vectors for the cell death detection. Briefly, trypsinized cells were collected, washed twice with ice-cold PBS, and resuspended in 200 μL binding buffer containing 5 μL FITC-conjugated Annexin V and 5 μL of propidium iodide. The staining sample was incubated at room temperature for 20 min, and 10,000 cells were immediately analysed using a FACSCalibur flow cytometer (BD Biosciences, USA).

### Immunofluorescence analysis and confocal microscopy

The cells were treated with equivalent amounts of sorafenib or DMSO for 24 h. Subsequently, the cells were fixed in 4% paraformaldehyde for 10 min and treated with 100% methanol for membrane permeabilization. The slides were blocked with 2% BSA (Sangon, China) at 37 °C for 30 min, fixed in 4% paraformaldehyde for 10 min, treated with antibodies diluted in 1% BSA overnight at 4 °C, and counterstained with DAPI. The immunofluorescence images were analysed under an Upright Metallurgical Microscope (Leica, Biberach, Germany) using the algorithm settings. The co-localization images were analysed by confocal laser scanning microscopy (CLSM, Leica, Germany). The positive rate (the number of positively stained cells/total cells) was then calculated.

### In vivo experiments

Athymic BALB/c nu/nu mice (4–6 weeks old) were purchased from SLAC Laboratory Animal Center (Changsha, China). The mice were weighed and randomly divided into eight groups (*n* = 5). To generate murine subcutaneous tumours, 0.5 * 10^6^ HepG2 cells transfected with control or HMGB1-specific shRNA were injected subcutaneously into the right armpit of the mice. For continuous stimulation in vivo, the mice were maintained under 12-h light/dark cycles with a regular diet of sorafenib (40 mg/kg body weight). The mice were treated for 21 days, and the tumour volumes were measured twice weekly. The volumes were calculated using the formula V = 1/ 2a^2^b, where a and b represent the shortest and longest diameters of the tumour, respectively. The mice were weighed twice weekly and sacrificed if they lost >20% of their body weight or appeared moribund or the tumour reached 1300 to 1500 mm^3^. The animals were euthanized 21 days post intervention, and the tumour tissues were harvested by resection and fixed in 10% formaldehyde for the immunohistochemistry or stored. The Animal Use Committee of Xiangya Hospital approved all animal treatment protocols, and all mice were treated humanely during the entire study period.

### Haematoxylin and eosin (HE) and IHC staining

After embedding in paraffin, the samples were cut into 10-μm slices and stained with HE. Some samples were cut into 4-μm slices, incubated with the HMGB1 antibody (Cell Signaling, 1:400), and subsequently incubated with a secondary antibody conjugated with streptavidin–biotin–peroxidase complex (Zhongshan Goldenbridge Biotechnology, Beijing, China). The colour reaction was developed using 3,3-diaminobenzidine tetrahydrochloride (Sigma-Aldrich).

### Statistical analysis

The quantitative data are presented as the means ± s.d of at least three independent experiments. The statistical analyses were performed using SPSS 19.0 software, and Student’s t-test was applied to compare the significant differences between two groups. All tests were two-sided, and differences were considered statistically significant at a *p*-value <0.05.

## Results

### HMGB1 is upregulated in response to sorafenib treatment in HepG2 cells

**Fig. 1:**
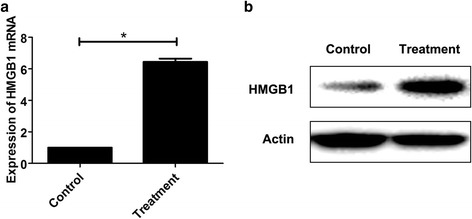
HMGB1 is upregulated in HepG2 cells following sorafenib treatment (*p < 0.05). HepG2 cells were treated with 5 μM sorafenib (Sor) or DMSO, and the HMGB1 expression was measured by (**a**) qPCR and (**b**) western blotting

The effects of sorafenib on the expression of HMGB1 were explored in the HCC cell line HepG2. The HMGB1 mRNA and protein levels were increased following a sorafenib treatment in the clinically relevant range (Fig. 1).

### Suppression of HMGB1 increases the sensitivity to sorafenib in HepG2 cells

**Fig. 2:**
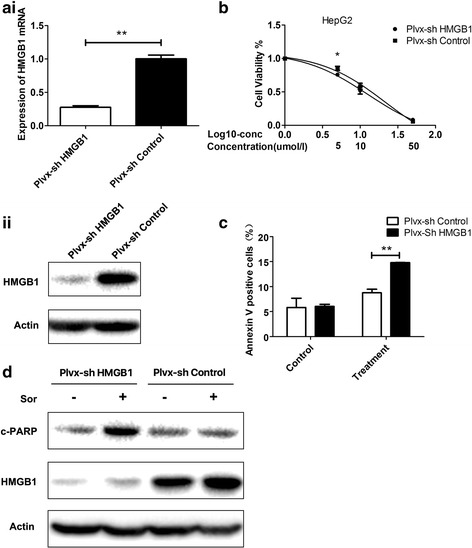
Suppression of HMGB1 increases the sensitivity to sorafenib in HepG2 cells (*p < 0.05; **p < 0.01). **Ai** qPCR and **Aii** western blotting results show that the expression of HMGB1 in HepG2 cells transfected with HMGB1 shRNA (Plvx-sh HMGB1) is significantly lower than that in sh-control HepG2 cells (Plvx-sh Control). **B** The impact of the HMGB1 knockdown on drug sensitivity at different sorafenib doses as determined by a CCK-8 assay. Plvx-sh HMGB1 cells showed an increased sensitivity to sorafenib at 5 μM. **C** Plvx-sh HMGB1 knockdown and Plvx-sh Control HepG2 cells were treated with 5 μM sorafenib (treatment) or the same volume of DMSO (control) for 48 h. Annexin V positive cells, consisting of both early and late apoptotic cells, were counted. **D** Expression levels of the apoptosis-related protein c-PARP were examined by western blotting

Sh-HMGB1 HepG2 and sh-control HepG2 cells were generated by transfection with HMGB1 shRNA and a control vector, respectively. The HMGB1 expression was significantly lower in the sh-HMGB1 HepG2 cells than in the control cells (Fig. 2A). According to the cell viability tests, the sh-HMGB1 HepG2 cells treated with sorafenib tended to be more sensitive to the drug than the sh-control cells (Fig. 2B). The apoptotic cells were quantified by determining the percentage of Annexin-V-positive cells 48 h following the sorafenib (5 μM) treatment using flow cytometry. Significantly higher levels of apoptosis were detected in the sh-HMGB1 cells than in the sh-control cells (Fig. 2C). Consistently, the expression of the apoptosis-related protein c-PARP increased following the sorafenib treatment in the HepG2 HMGB1 knockdown cells (Fig. 2D). Altogether, HMGB1 played a protective role in HepG2 cells following exposure to sorafenib.

### Overexpression of HMGB1 increases resistance to sorafenib in HepG2 cells

**Fig. 3:**
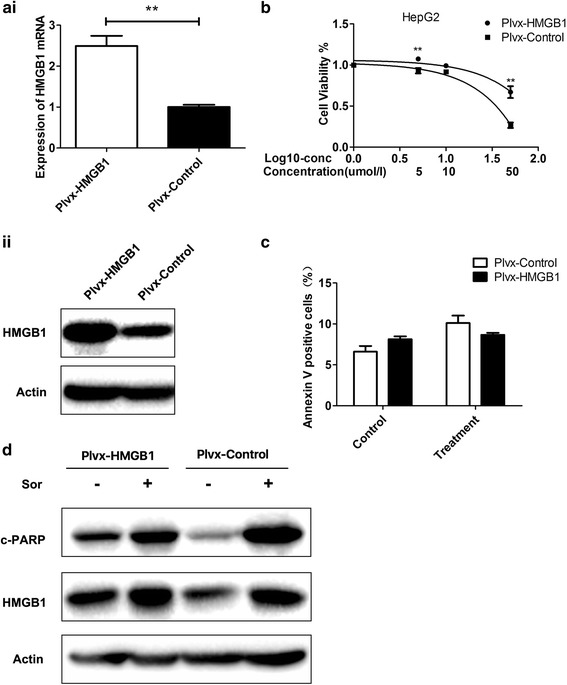
Overexpression of HMGB1 increases the resistance to sorafenib in HepG2 cells. (*p < 0.05; **p < 0.01). **Ai** qPCR and **Aii** western blot analyses show the overexpression of HMGB1 in Plvx-HMGB1 cells compared with that in Plvx-control HepG2 cells. **B** HMGB1 overexpression affects drug sensitivity at different sorafenib doses as determined by a CCK-8 assay. **C** Plvx-HMGB1 cells and Plvx-control HepG2 cells were treated with 5 μM sorafenib (treatment) or DMSO at same volume (control) for 48 h. The percentage of Annexin V positive cells was measured as an indicator of early and late apoptotic cells. **D** c-PARP was examined to measure the apoptosis levels in HMGB1-overexpressing HepG2 cells and Plvx-Control HepG2 cells

To further explore the role of HMGB1 in the regulation of sorafenib sensitivity, we overexpressed HMGB1 in HepG2 cells. HMGB1 overexpression vectors were transfected into HepG2 cells, resulting in a significant increase in HMGB1 protein and mRNA in the resultant Plvx-HMGB1 HepG2 cell line (Fig. 3A). Furthermore, according to the cell viability assays, the Plvx-HMGB1 HepG2 cells were less sensitive to the sorafenib treatment than the control cells (Fig. 3B). According to the flow cytometry analysis, compared to the HMGB1 overexpression group, the Plvx-Control group showed a slight but insignificant increase in apoptosis following the sorafenib intervention (Fig. 3C). Compared with the Plvx-HMGB1 HepG2 group, the Plvx-control HepG2 group exhibited a greater increase in the c-PARP levels, indicating that the HMGB1 overexpression could reduce sorafenib-induced apoptosis (Fig. 3D).

### HMGB1 translocates from the mitochondria to the cytoplasm following sorafenib treatment

**Fig. 4:**
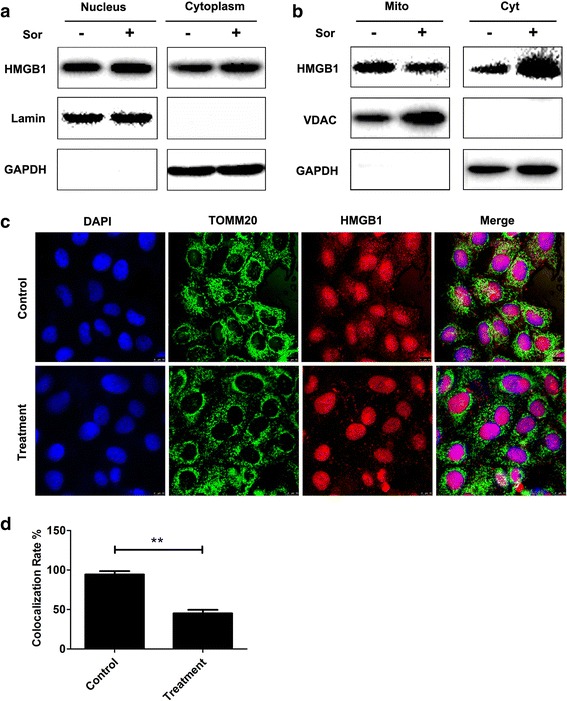
HMGB1 translocates from the mitochondria to the cytoplasm after sorafenib treatment. HepG2 cells were untreated or treated with 5 μM sorafenib for 48 h. **a** Nuclear and cytoplasmic proteins were separated, and the expression of HMGB1 in both fractions was examined by western blotting. **b** HMGB1 expression in extracted mitochondria (Mito) and the remaining cytoplasm without the mitochondria (Cyt) was examined. **c** HepG2 cells were untreated (Control) or treated with 5 μM sorafenib for 48 h (Treatment), and stained for HMGB1 (red), DAPI (blue), and tomm20 (green) to visualize the mitochondria. Cells were examined by confocal microscopy (magnification, ×630). **d** Colocalization of HMGB1 with mitochondria in HepG2 cells was analysed in images from three independent experiments (**p < 0.01)

To further examine the relationship between HMGB1 and sorafenib resistance, the HMGB1 distribution in HepG2 cells exposed to sorafenib was examined. The total nuclear protein was separated from the cytoplasmic protein, and no significant change in the HMGB1 expression was observed in either fraction following the sorafenib treatment (Fig. 4a). The amount of mitochondrial HMGB1 in the HepG2 cells decreased following the sorafenib exposure, while the amount of cytosolic HMGB1 increased (Fig. 4b). Therefore, HMGB1 translocated from the mitochondria to the cytosol in the HepG2 cells following the sorafenib treatment. In the HepG2 cells, the Tomm20-stained mitochondria and HMGB1 co-localized (Fig. 4c). The co-localization of the Tomm20-stained mitochondria and HMGB1 significantly decreased following a 48-h sorafenib treatment (Fig. 4d).

### Suppression of HMGB1 increases the sensitivity to sorafenib in vivo

**Fig. 5:**
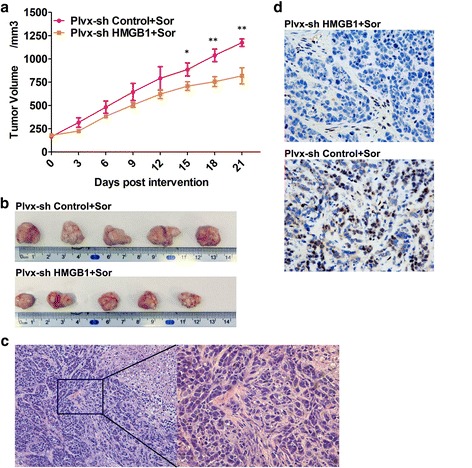
Suppression of HMGB1 increases the sensitivity to sorafenib in vivo (*p < 0.05; **p < 0.01). HepG2 cells stably expressing sh-HMGB1 or sh-control were injected subcutaneously into the flanks of nude mice administered sorafenib (40 mg/kg/day for 21 days). **a** Tumour growth curves in the different groups were recorded. **b** Images of dissected tumours from each group (*n* = 5) at the end of the experiment. **c** HE staining of the tumours. **d** Immunohistochemical analysis and quantitative evaluation of xenograft tumour tissues using HMGB1 antibodies

To examine the effects of HMGB1 in sorafenib resistance in vivo, BALB/c nu/nu mice were inoculated with HepG2 cells transfected with HMGB1-specific shRNA or control shRNA. Three weeks after the subcutaneous tumour implantation, the tumours reached 100 to 200 mm^3^ in size, and the athymic mice were treated with 40 mg/kg sorafenib via a p.o. gavage daily. The average tumour volume in the group of mice injected with sh-HMGB1 HepG2 was smaller than that in the group of mice injected with the sh-control HepG2 cells at all time points. Therefore, the tumour inhibition effect of sorafenib was better in the sh-HMGB1 group (Fig. 5a). The tumours were detached from the mice in each group at the end of the experiment (Fig. 5b). HE staining was performed to examine the tumour, revealing that the abnormal cells were distributed in small nests or cord-like shapes, and some necrotic cells could be observed (Fig. 5c). The HMGB1 immunohistochemical staining (IHC) of the tumour specimens was performed to determine the HMGB1 expression levels in the Plvx-sh HMGB1 and Plvx-HMGB1 groups in vivo (Fig. 5d). Altogether, HMGB1 plays a critical role in the regulation of sorafenib resistance in HepG2 cells in vivo.

## Discussion

Sorafenib is a multi-kinase inhibitor with the anticancer effects of suppressing tumour proliferation and angiogenesis and inducing apoptosis. Sorafenib suppresses tumour proliferation by inhibiting the mitogen-activated protein kinase (MAPK) family members Raf-1 and B-Raf. In addition, sorafenib acts by blocking receptors, including VEGFR and PDGFR, leading to the inhibition of angiogenesis [30]. Multiple signalling pathways may contribute to the acquisition of sorafenib resistance. Notably, the epithelial-mesenchymal transition and the balance between apoptosis and autophagy have been shown to play prominent roles in producing resistance to sorafenib [31–35]. Additionally, the activation of an alteration or escape from the Ras/Raf/MAPK pathway in tumour cells likely contributes to chemoresistance [36]. However, the phosphatidylinositol 3-kinase/Akt (PI3K/Akt) signalling pathway, which is involved in cell survival and death in human malignancies, is suggested to be a major contributing factor [30, 32].

HMGB1 is a versatile protein in tumour biology and cancer therapy [24]. The role of HMGB1 in the intervening chemotherapy response could be associated with its sub-cellular localization and the corresponding biological events it mediates. Intracellular HMGB1 induces cell proliferation, migration, and invasion via the MAPK and/or PI3K/Akt signalling pathways in malignancies, including myofibroblasts and human cutaneous squamous cell carcinoma [37]. The activation of Akt leads to the reduced phosphorylation of proapoptotic proteins. HMGB1 interacts with MAPK and/or PI3K/Akt to promote cell proliferation and autophagy and plays roles in inflammation. Feng et al. revealed that the activation of the PI3K/Akt signalling pathways mediated HMGB1-induced proliferation in MMC cells [38]. MAPK positively regulates HMGB1-mediated autophagy [23]. In turn, ERK, which is a class of MAPKs, has been shown to promote HMGB1 release in inflammatory models [39].

Intracellular HMGB1 was thought to be involved in both apoptosis and autophagy. HMGB1 has been suggested to normally have anti-apoptotic effects in cancers, but paradoxical roles in promoting apoptosis were identified in cardiomyocytes and mouse embryonic fibroblasts [16, 40–45]. Furthermore, nuclear HMGB1 indirectly regulates heat shock protein b-1 (HSPB1 or HSP27), which is an important intracellular factor that plays roles in maintaining the quality of mitochondria [46] and may indirectly affect mitochondria-mediated apoptosis. HMGB1-mediated autophagy, resulting in aggravating chemoresistance, has been demonstrated in cancers, including osteosarcoma, lung cancer, leukaemia, and gastric cancer [47–51]. Cytosolic HMGB1 directly binds Beclin1 and subsequently disrupts the Bcl2-Beclin1 interaction, leading to the formation of Beclin-1-mediated autophagosomes [52].

Extracellular HMGB1 may affect chemoresistance through several methods. The classical HMGB1-RAGE signalling axis and HMGB1-TLR4 pathways have been suggested to play important roles in cancer [53]. Because it is an important DAMP, HMGB1-mediated immunogenic cell death enhances the effects of chemotherapy by promoting tumour cell death. HMGB1 release from cells was demonstrated to promote pancreatic tumour growth by binding RAGE and eventually regulating mitochondrial bioenergetics [47]. Additionally, the interaction between HMGB1 and TLR4 mediates anti-cancer immunity during radio- and/or chemotherapy [54].

Because no previous reports detailed the role of HMGB1 in the HCC chemotherapeutic response, our data suggest for the first time that a knockdown of HMGB1 restores the sensitivity to sorafenib in vitro in HepG2 cells and in vivo. Furthermore, we found that HMGB1 translocates from the mitochondria to the outside cytoplasm in HepG2 cells following treatment with sorafenib. Our findings led to additional questions. Does HMGB1 function in all HCC cell lines or drug-resistant cell lines? Is the phenomenon of mitochondrial HMGB1 translocation associated with the role of HMGB1 in drug sensitivity? What is the function of mitochondrial HMGB1 and cytosolic HMGB1? How does HMGB1 promote sorafenib resistance in HepG2 cells?

**Fig. 6:**
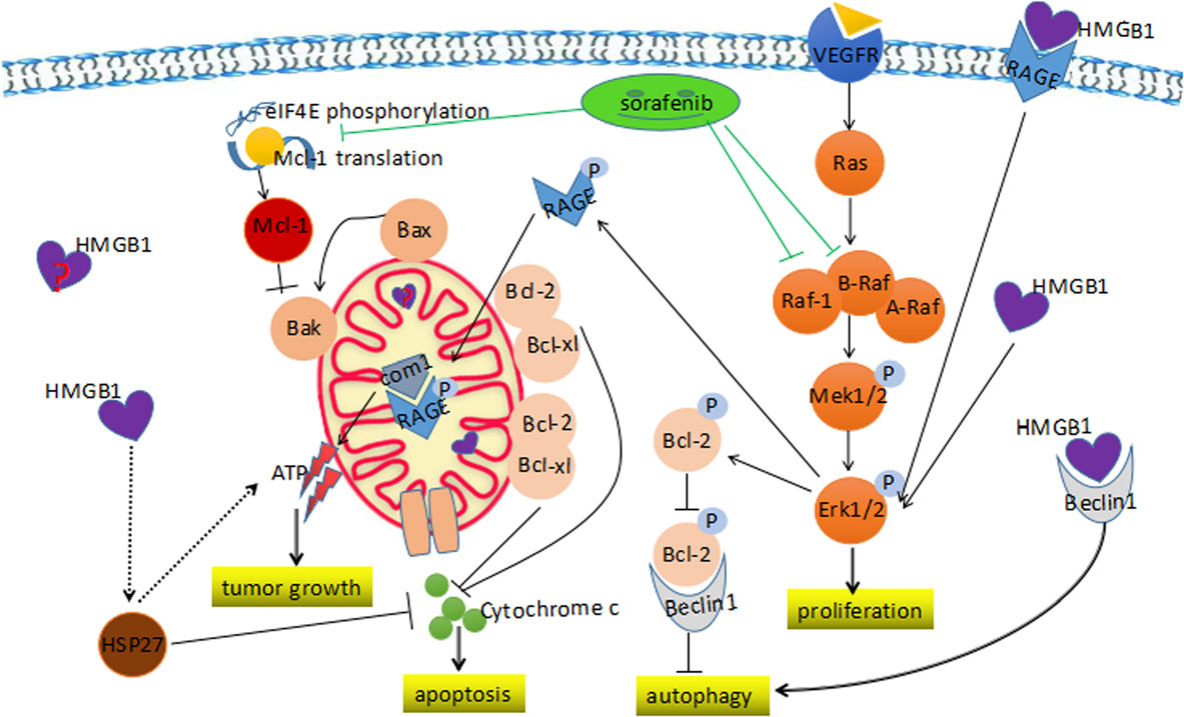
Potential mechanism of HMGB1-induced sorafenib resistance in HCC. Sorafenib targets Raf-1, B-Raf, and eIF4E phosphorylation, which leads to the inhibition of proliferation and induces apoptosis. However, intracellular HMGB1 promotes the downstream MAPK cascade through Erk1/2 phosphorylation. Then, phospho-Erk attenuates the competition between phospho-Bcl-2 and HMGB1 binding to Beclin 1. Nuclear HMGB1 and HSP27 maintain mitophagy and act as co-factors to block apoptosis. Meanwhile, through the HMGB1/RAGE axis, extracellular HMGB1 indirectly promotes tumour growth by accelerating ATP production

While we do not have the answers to these questions, these findings have helped us ask better questions. To provide insight from previous studies, the roles of HMGB1 in multiple biological events and the functions of sorafenib as a tyrosine kinase inhibitor are summarized in a figure. This overview offers certain insights into the mechanism by which HMGB1 potentially contributes to sorafenib resistance and the potential role of mitochondrial HMGB1 in this process (Fig. 6) [30, 47, 55–57].

This study has certain limitations. The effects were observed in a single HCC cell line, i.e., HepG2, and may be insufficient to prove that HMGB1 plays a role in sorafenib resistance in HCC. Further studies are necessary to determine whether HMGB1 regulates sorafenib resistance in different HCC cell lines. Meanwhile, elucidating the functions of cytosolic HMGB1 and mitochondrial HMGB1 may provide new insights into the different roles of HMGB1.

## Conclusions

HMGB1 is involved in sorafenib resistance in HepG2 cells and in vivo. Furthermore, this is the first report to show that HMGB1 downregulation promotes the sensitivity to sorafenib. The translocation of HMGB1 from the mitochondria to the cytoplasm following sorafenib exposure requires further investigation to identify its association with the sensitivity of HepG2 to sorafenib. Our study provides new insights into the relationship between HMGB1 and sorafenib resistance in HepG2 cells. While further studies are necessary, we postulate that HMGB1 provides a new mode for regulating sorafenib resistance and could serve as a novel potential target to stratify patients suitable for sorafenib treatment or contribute to combination drug use in HCC.

## Additional file

Additional file 1: FCM of Figs. 2C and 3C. The Excel spreadsheet shows the flow cytometry results of the shHMGB1 HepG2, HMGB1-overexpressing HepG2 cells and corresponding control cells with or without the sorafenib treatment. (CSV 1 kb)

## Abbreviations

CCK-8: Cell counting kit-8
DAMPs: Damage-associated molecular patterns
DMEM: Dulbecco’s modified eagle medium
EMT: Epithelial mesenchymal transition
FBS: Foetal bovine serum
HCC: Hepatocellular carcinoma
HE: Haematoxylin and eosin
HMGB1: High mobility group box 1
HSPB1: Heat shock protein B-1
IHC: Immunohistochemistry
MAPK: Mitogen-activated protein kinase
PDGFR: Platelet-derived growth factor receptor
PI3K/Akt: Phosphatidylinositol 3-kinase/Akt
RAGEs: Receptor for advanced glycation end products
TLRs: Toll-like receptors
VEGFR: Vascular endothelial growth factor receptor
